# The Role of Renal Denervation in HFpEF

**DOI:** 10.3390/jcm14124115

**Published:** 2025-06-10

**Authors:** Dawood Jamil, Sanaullah Mojaddedi, Patrick Kollman, Najeebullah Bangash, Omar Sami Abdelhai, Yazeed Aburuman, Amir S. Lotfi

**Affiliations:** 1 Department of Internal Medicine, Henry Ford Hospital, Detroit, MI 48202, USA; oabdelh1@hfhs.org; 2 College of Medicine, University of Central Florida, Graduate Medical Education, Orlando, FL 32827, USA; sanaullah.mojaddedi@hcahealthcare.com; 3 Internal Medicine Residency Program, HCA Florida North Florida Hospital, Gainesville, FL 32605, USA; 4 School of Medicine, Wayne State University, Detroit, MI 48202, USA; pkollman@wayne.edu; 5 Department of Cardiology, Corewell Health Dearborn Hospital, Dearborn, MI 48124, USA; najeebullah.bangash@corewellhealth.org; 6 Department of Internal Medicine, Saint Michael’s Medical Center, Newark, NJ 07102, USA; yazeedfawwaz@gmail.com; 7 Department of Cardiology, Baystate Medical Center, Springfield, MA 01199, USA; amir.lotfi@baystatehealth.org

**Keywords:** renal denervation, heart failure with preserved ejection fraction, interventional cardiology approaches, cardiac remodeling

## Abstract

Heart failure with preserved ejection fraction (HFpEF) is a complex and heterogeneous clinical syndrome characterized by signs and symptoms of heart failure despite normal or near-normal ejection fraction. It is a debilitating chronic disease that affects millions of people worldwide, and due to the paucity of evidence-based pharmacological treatments for HFpEF, nonpharmacological approaches as potential therapeutic alternatives are of growing interest. As a result, renal denervation (RDN), initially developed as a therapeutic tool for resistant hypertension, has become an area of active clinical interest. RDN is a catheter-based procedure that targets the renal sympathetic pathways, aiming to reduce neurohormonal activation and mitigate maladaptive cardiac remodeling. Preclinical studies in animal models have demonstrated that RDN can improve cardiac and vascular fibrosis, reduce renal inflammation, control hypertension, and alleviate endothelial dysfunction. Recent clinical studies have further highlighted the potential benefits of RDN in patients with HFpEF and uncontrolled hypertension. In this review, we aim to outline the pathophysiology of HFpEF and demonstrate the complex clinical interplay involved in how RDN impacts the heart. Moreover, we discuss the present status of clinical studies on RDN and explore its therapeutic potential as a viable treatment for HFpEF.

## 1. Introduction

HFpEF is a subset of heart failure characterized by an ejection fraction above 50% [1,2,3]. It accounts for approximately half of all cases of heart failure and is a debilitating chronic disease that affects 32 million individuals worldwide [1,4,5]. Similar to heart failure with reduced ejection fraction (HFrEF), it presents symptoms such as shortness of breath, congestion, and fatigue; however, it features a distinct and heterogeneous pathophysiology that remains incompletely understood [6].

Emerging evidence suggests that pro-inflammatory conditions such as metabolic syndrome and diabetes may play a significant role in HFpEF pathophysiology, wherein they lead to myocardial fibrosis, hypertrophy, and, ultimately, diastolic dysfunction [1,7,8]. Alongside these inflammatory mechanisms, there has been increasing interest in the contribution of neurohormonal activation, particularly given its role in HFrEF; however, studies investigating the role of SNS overactivation in HFpEF have demonstrated conflicting findings.

Despite advances in our understanding of the multifaceted pathophysiology of HFpEF, effective treatments remain scarce, with the current recommended management of HFpEF primarily consisting of pharmacological therapies. Sodium–glucose co-transporter 2 (SGLT2) inhibitors serve as one of the cornerstones in the management of HFpEF, as demonstrated in the Empagliflozin Outcome Trial in Patients with Chronic Heart Failure with Preserved Ejection Fraction (EMPEROR Preserved), which showed that empagliflozin decreased hospitalizations by 29% and cardiac death by 21% compared to a placebo [9]. Similarly, the Treatment of Preserved Cardiac Function Heart Failure with an Aldosterone Antagonist (TOPCAT) trial revealed that spironolactone significantly decreased hospitalizations in North American HFpEF patients compared to a placebo [10,11]. These limitations have sparked growing interest in nonpharmacological strategies as complementary or alternative treatment options [12].

RDN is a catheter-based therapy initially developed for resistant HTN that targets renal sympathetic nerves to reduce sympathetic overactivity [13]. In this review, we provide a detailed discussion of the pathophysiology of HFpEF and recent studies on RDN in HFpEF, including its effects on functional outcomes, cardiac biomarkers, and structural cardiac changes in HFpEF patients. We conclude with a discussion on the current role of RDN in HFpEF and a critical evaluation of the existing evidence.

## 2. Modalities of Renal Denervation and Their Role in Hypertension Management

Three modalities are currently used in RDN procedures: radiofrequency ablation (RFA), ultrasound, and neurotoxin injection [14]. In the RFA approach, electrodes are placed in the renal arterial vasculature, and a medium-frequency alternating current generates heat that damages surrounding nerves while preserving the arterial wall [15,16]. Ultrasound RDN systems involve a small ultrasound-emitting transducer placed in the renal artery, which is then surrounded by a water-pressurized balloon that cools and protects the arterial intima while heat is applied to the surrounding nerves [15]. In neurotoxin-mediated RDN, microneedles are used to inject ethanol directly into the perivascular space.

Numerous clinical trials have evaluated the effect of RDN on blood pressure in patients with hypertension, with mixed yet promising results. The first large-scale sham-controlled Randomized Controlled Trial (RCT), the SYMPLICITY HTN-3 trial, failed to demonstrate an improvement in systolic blood pressure (SBP) following RFA RDN in patients with resistant hypertension [17]. In the subsequent SPYRAL-HTN ON MED and SPYRAL-HTN OFF MED pilot studies, RFA procedures were shown to decrease SBP compared to sham controls by 7.4 mmHg and 5 mmHg for patients on and off medical antihypertensive therapy, respectively [18,19]. In contrast, the 2023 SPYRAL-HTN ON MED expansion and the 2019 REDUCE-HTN: REINFORCE trials both failed to demonstrate a significant SBP reduction with RFA procedures over sham-controlled groups [16].

Trials using ultrasound ablation have shown more promise, with the RADIANCE TRIO and RADIANCE SOLO trials demonstrating a 4.5 mmHg and 6.3 mmHg reduction in SBP, respectively, compared to sham-controlled groups with patients who were on and off of antihypertensive medications [16,20,21].

TARGET-BP 1 and TARGET-BP OFF MED trials evaluated the efficacy of neurotoxin (alcohol) denervation. The TARGET-BP 1 trial found a 3.2 mmHg decrease in SBP following RDN, while the TARGET-BP OFF MED trial found no significant difference between RDN and non-RDN groups [16,19,22]. A 2024 meta-analysis including multiple RDN modalities concluded that RDN safely and significantly lowers SBP in settings of uncontrolled hypertension both on and off medical antihypertensive therapy [23].

Selective RDN, where sympathetic renal nerves are more precisely identified and targeted, is a promising new direction in RDN techniques. A large-scale sham-controlled RCT, the SMART trial, is ongoing and aims to examine the efficacy of renal nerve mapping and selective RDN. At 6-month follow-up, selective RDN successfully decreased the medication burden of hypertensive patients while achieving better blood pressure control than previous RDN trials, where renal arterial nerves were globally rather than selectively ablated [24].

## 3. Pathophysiology of HFpEF and the Impact of Renal Denervation

HFpEF is a complex syndrome with multiple underlying causes and pathophysiologic mechanisms driven by both cardiac and non-cardiac processes. Historically, arterial hypertension has been implicated as the main culprit, causing increased afterload that leads to concentric LV hypertrophy and myocardial stiffening. Over time, our understanding has evolved, with new evidence indicating that HFpEF is driven by a combination of cardiovascular and non-cardiovascular determinants, including hypertension, obesity, diabetes mellitus, chronic obstructive pulmonary disease (COPD), and metabolic syndrome. These non-cardiovascular determinants contribute to systemic inflammation and dysfunction of microvascular and endothelial cells [25].

In addition to these mechanisms, the overactivation of the Sympathetic Nervous System (SNS) has long been debated in HFpEF disease states. While the role of the SNS is well-documented in HFrEF, the evidence has been inconsistent. Several studies have attempted to quantify SNS activity in HFpEF using various modalities. SNS activity can be measured directly by plasma or urinary norepinephrine (NE) levels and muscle sympathetic nerve activity (MSNA), indirectly via Heart Rate Variability (HRV), or, secondly, visualized via 123I-metaiodobenzylguanidine (MIBG) scintigraphy.

A study by Arora et al. found that patients with diastolic dysfunction exhibit reduced HRV, indicating an altered sympathetic–parasympathetic balance [26]. Furthermore, Grassi et al. found that patients with diastolic dysfunction exhibited significantly greater sympathetic nerve activity in response to blood pressure increases compared to controls despite no difference in plasma norepinephrine levels [27]. In another study, 20 patients with HFpEF underwent arterial and coronary sinus sampling, which revealed elevated arterial NE levels and a significant trans-cardiac NE gradient, indicative of heightened cardiac sympathetic tone [28]. Other studies found no differences in serum Norepinephrine (NE) levels between HFrEF and HFpEF groups [29,30].

While these studies provide evidence for increased sympathetic tone in HFpEF, their interpretation is limited by heterogeneous study designs, limited patient cohorts, and varying inclusion criteria. Furthermore, surrogate markers such as HRV and serum NE levels are crude measures of sympathetic activity, and other factors, including renal dysfunction, can influence serum NE levels. Another notable limitation is that it remains unclear whether the present research demonstrating increased SNS activity is simply a byproduct of HFpEF, a consequence of HFpEF, or a result of its multiple comorbidities.

This uncertainty surrounding sympathetic activation extends to pharmacologic strategies, such as beta-blockers (BBs), which are used in an estimated 50–80% of patients with HFpEF and have shown questionable benefits [31]. A secondary analysis of the TOPCAT Trial showed that beta-blockade was associated with a higher risk of heart failure hospitalization [32]. Additionally, subgroup analysis of the CIBIS-ELD trial showed similar tolerability between the HFrEF and HFpEF groups; however, only the HFrEF group demonstrated improvements in clinical parameters and left ventricular function [33]. A subgroup analysis of the DELIVER trial showed that in patients with HFmrEF or HFpEF, its use was not associated with an increased risk of cardiovascular mortality or worsening heart failure [34]. Moreover, in a recent meta-analysis, beta-blockade was associated with a reduction in all-cause mortality in HFpEF; however, most of the data was derived from observational studies [35]. When considered as a whole, beta-blockade has shown some beneficial effects, but these may be offset by its negative chronotropic effects. Patients with HFpEF often have limited stroke volume reserve; thus, beta-blockade can lead to an impaired chronotropic response, which can ultimately worsen outcomes [32,36].

These studies’ mixed findings underscore the well-known heterogeneity of HFpEF, wherein different phenotypes may have varying degrees of neurohormonal activation. Recent studies show that hemodynamic profiles can vary across the spectrum of HFpEF based on LVEF, wherein patients with an EF < 60% have features that resemble HFrEF with impaired ventriculo-arterial coupling and reduced contractility, whereas those with EF > 60% exhibit a hypercontractile state with higher afterload and a limited preload reserve [37].

**Figure 1:**
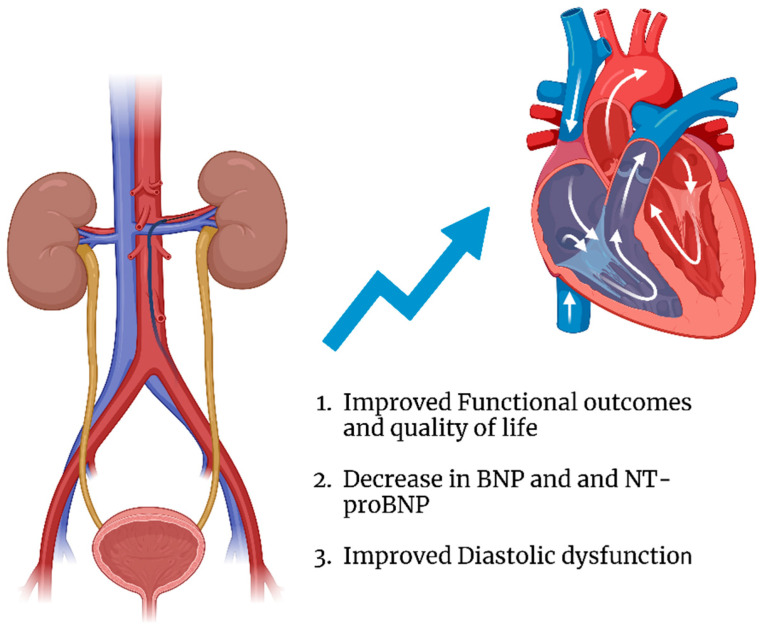
Illustration demonstrating the impact of RDN on HFpEF.

Although studies regarding SNS overdrive in HFpEF have shown mixed results, given its efficacy in patients with uncontrolled HTN, this prompted investigation into whether RDN could offer similar benefits in HFpEF. As illustrated in Figure 1, observational studies indicate that RDN may be associated with improvements in diastolic function, reductions in natriuretic peptide levels, and enhanced functional status, areas that will be further examined in this paper.

## 4. Clinical Studies on Renal Denervation in HFpEF

### 4.1. Functional Outcomes and Quality of Life

Quality of life (QOL) and functional outcomes are integral to treating patients with HFpEF, as they can serve as prognostic indicators and measures of therapeutic success [38]. Quality of life is typically assessed using validated questionnaires such as the Kansas City Cardiomyopathy Questionnaire (KCCQ) or the Minnesota Living with Heart Failure Questionnaire (MLHFQ) [39]. Functional capacity, on the other hand, indicates the patient’s ability to perform daily tasks and is assessed via peak oxygen consumption (peak VO2), the 6 min walk test (6MWT), and New York Heart Association (NYHA) classes.

The RDT-PEF study by Patel et al. was the first RCT to evaluate the effects of RDN in patients with HFpEF [40]. It was a phase II, prospective single-center RCT in which 25 patients with HFpEF were randomized into an RDN group using a SymplicityTM catheter and a medical therapy group. The study aimed to evaluate biomarkers, cardiac remodeling, functional status, and QOL via the MLWHFQ and VO2 peak [40]. It found that at three months post-RDN, there was a statistically significant improvement in peak VO2, with 56% of patients in the RDN group showing improvement compared to 13% in the medical therapy group (*p* = 0.025); however, these effects were not sustained at 12 months [40]. Similarly, quality of life assessed via the MLWHFQ showed no improvement at 12 months; however, it is important to note that this study was underpowered to detect a difference due to difficulties in patient recruitment stemming from stringent inclusion criteria despite nationwide screening [40].

Conversely, a retrospective analysis by Kresoja et al. evaluated sixty-four patients with uncontrolled arterial hypertension (aHT) who underwent RDN between 2011 and 2018, including 99 with HFpEF, and had differing findings [41].

It was a single-center study wherein echocardiographic and cardiac magnetic resonance (CMR) imaging were performed, and patients were stratified into HFpEF and non-HF failure groups based on the European Society of Cardiology guidelines. At baseline, 65 out of 99 patients in the HFpEF group had an NYHA functional class ≥II, compared to 31 out of 65 patients in the non-HF group [41]. Following RDN, patients with HFpEF were noted to have significant improvement in their NYHA class at 6 months compared to patients without heart failure, with 21% of patients improving after at least 1 class (*p* < 0.001) [41].

Supporting these findings, Rommel et al. (2023) conducted a retrospective cohort study involving 60 patients who underwent RDN from 2015 to 2018, which included 30 with HFpEF and 30 with resistant aHT as controls [42]. At baseline, 100% of the patients with HFpEF were classified as NYHA class ≥II. However, after RDN, this percentage significantly decreased to 63% (*p* < 0.001). In contrast, the control group experienced only a slight reduction in the proportion of patients with an NYHA Class ≥II, from 50% to 40%, which was not statistically significant (*p* = 0.13) [42].

Clinically, the application of RDN in HFpEF has yielded mixed results regarding functional outcomes and QOL. The RDT-PEF study by Patel et al. was a pioneering RCT that evaluated the role of RDN in HFpEF. Although it was underpowered, initial results showed promise, with improvements in VO_2_ peak at three months [40]. However, these benefits were not sustained. Subsequent studies by Kresoja et al. and Rommel et al. yielded more favorable outcomes, demonstrating ongoing reductions in NYHA classes.

### 4.2. Cardiac Biomarkers

Cardiac biomarkers, such as BNP and NT-proBNP, play vital roles in diagnosing, stratifying, and monitoring the progression of heart failure [43,44]. In patients with HFpEF, elevated levels of NT-proBNP can indicate increased ventricular wall strain and heightened left ventricular end-diastolic pressure, both of which are key indices in HFpEF [45]. Additionally, it can serve as a strong predictor of mortality, as demonstrated in a study where a 2.7-fold increase in NT-proBNP corresponded to a 2.14-fold hazard ratio for mortality [46]. Investigating the effect of RDN on NT-proBNP/BNP is therefore essential, as it can provide valuable insights into the role of RDN in improving cardiac outcomes and reducing mortality.

The RDT-PEF study by Patel et al. evaluated the effects of RDN on BNP [40]. It defined a clinically relevant change of Δ BNP of 50 ng/L (SD 130). At baseline, patients in the RDN group had a BNP of 210 (137–354), while the control group had a BNP of 149 (99–205) [40]. At the end of the 12-month study, no statistically significant change in BNP was observed in the HFpEF or control groups [40].

The study by Kresoja et al. evaluated 99 patients with HFpEF who underwent RDN compared to 65 without [41]. NT-proBNP was obtained prior to RDN and within 6 months post-RDN. At baseline, patients with HFpEF had a significantly higher NT-proBNP than their non-HF counterparts at 301 versus 65 ng/L (*p* = 0.011) [41]. Post-procedure, there was a significant decrease in NT-proBNP in patients with HFpEF, with an average reduction of 24% (*p* = 0.011); in contrast, patients without heart failure had a 9% increase in NT-proBNP (*p* = 0.011) [41].

The study by Rommel et al. demonstrated similar results. At baseline, patients in the HFpEF cohort had a median NT-proBNP of approximately 315 ng/L compared to a median of approximately 67 ng/L in the control group [42]. Post-RDN, the median NT-proBNP in the HFpEF group decreased to approximately 260 ng/L, while the control group slightly increased, with a median NT-proBNP of approximately 75 ng/L (*p* < 0.001) [42].

Vogt et al. assessed long-term outcomes in 70 patients using the HFA-PEFF (Heart Failure Association Pre-test Assessment of HFpEF) score, a diagnostic tool developed by the European Society of Cardiology to diagnose HFpEF [47]. The scoring system consists of three domains: functional domains, morphological domains, and biomarker domains, each of which is assigned points [47]. An NT-proBNP > 220 pg = mL or BNP > 80 pg = mL confers 2 of 5 possible points for patients without atrial fibrillation [47]. Further, 21 of the 70 patients had HFpEF, with a baseline score of 5.48 ± 0.51 points and a score of 5 with a specificity of 91% for diagnosing HFpEF [48]. At the 9-year follow-up, this significantly decreased to 4.33 ± 1.53, driven primarily by morphological and biomarker categories, with the biomarker category decreasing from 1.52 ± 0.52 to 0.90 ± 0.63 (*p* < 0.01) [48].

The effect of RDN on cardiac biomarkers, specifically BNP and NT-proBNP, has produced conflicting findings. While the RDT-PEF did not yield substantial differences in BNP 12 months post-procedure, more recent studies by Kresoja et al., Rommel et al., and Vogt et al. highlight the beneficial effect of RDN on cardiac biomarkers, in which there was a notable decline in biomarker levels [41,42,48]. These findings align with the observed beneficial effect that RDN has on cardiac remodeling and diastolic measures.

### 4.3. Structural Cardiac Changes

HFpEF is a heterogeneous syndrome that accounts for approximately 50% of heart failure cases [49]. It is characterized by structural remodeling wherein patients exhibit diastolic dysfunction, elevated filling pressures, and increased ventricular hypertrophy and fibrosis [50].

Patel et al. performed the first RCT by looking at the role of RDN in patients with HFpEF. The trial enrolled 25 patients and was thus underpowered to detect a difference, as a sample size of 36 patients would be required for 85% power, making it susceptible to type 2 errors [40]. At three months, there was an improvement in the RDN group versus the control group concerning E/e′ (31% vs. 13%, *p* = 0.04); however, these effects were not sustained at twelve months [40].

Kresoja et al. did note improvements in diastolic properties in the HFpEF group compared to their non-heart failure counterparts [41]. At baseline, patients in the HFpEF group had a higher left ventricular ejection fraction (LVEF) at 64% versus 60%, with no statistically significant difference in ejection fraction post-RDN in either group [41]. Furthermore, the E/E′ ratio decreased significantly in the HFpEF group by 1.0 (*p* = 0.043) versus no change in the non-heart failure group (*p* = 0.90) [41]. Similarly, there was a modest decrease in Emax by 0.03 from a baseline of 0.86 (*p* = 0.015) in the HFpEF group [41].

Vogt et al. provided long-term data on cardiac remodeling post-RDN. At baseline, 17 (81%) patients in the HFpEF group had significant LV hypertrophy with a Left Ventricular Mass Index (LVMI) ≥149/122 g/m^2^; this figure decreased substantially to only four (19%) at 9-year follow-up [48]. This was accompanied by a concurrent reduction in significant LA dilation (>34 mL/m^2^), which reduced from 12 (57.1%) to 8 (38.1%) [48].

Zamani et al. also demonstrated improved diastolic dysfunction post-RDN in their study. He analyzed 22 patients with resistant hypertension and HFpEF [51]. Sixteen patients underwent RDN, and six controls received optimal medical therapy (OMT) [51]. Both groups had an EF ≥ 50% with elevated global longitudinal strain (GLS) ≥18% at baseline [51]. At 6-month follow-up, GLS improved in the RDN group, increasing in magnitude from −14.21% ± 3.19 to −17.17% ± 3.1 (*p* = 0.007), versus no change in the OMT group [51]. Moreover, there was a non-statistically significant reduction in Left Ventricular Mass (LVM) in the RDN group (58.55 g/m^2^ ± 11.37 vs. 55.46 g/m^2^ ± 12.76; *p* = 0.085) with no change in the OMT group [51].

The mechanism by which RDN exerts its therapeutic effect remains incompletely understood. However, many of these studies indicate that RDN confers favorable effects on cardiac remodeling and improves key indices such as GLS, LVH, and diastolic function. Table 1 provides a detailed summary of these findings, highlighting study type, cohort, endpoints, and results.

## 5. Discussion

Over the past few decades, many effective treatments have been developed for HFrEF and HFmrEF, which have led to improved clinical outcomes in these cohorts [52,53,54]. However, despite the multiplicity of effective treatments for HFrEF and HFmrEF, the majority of pharmacologic therapies have failed to show clinical benefit in HFpEF patients, underscoring the need for novel therapeutic interventions [55,56].

With the emergence of interventional cardiology and the proliferation of minimally invasive catheter-based interventions, RDN was explored in the early 2000s for patients with uncontrolled hypertension refractory to multiple antihypertensives. Initial trials such as SIMPLICITY-3-HTN demonstrated minimal antihypertensive effects [17]. Over time, operators gained greater experience with RDN, as evidenced by recent studies showing the enhanced efficacy of RDN, with sustained reductions in blood pressure [57]. This eventually garnered endorsements from the European Society of Cardiology Council on Hypertension and the European Association of Percutaneous Cardiovascular Interventions for RDN as an adjunct intervention for uncontrolled resistant hypertension [57,58].

Sympathetic nervous system overdrive plays an integral role in the pathogenesis of multiple cardiovascular disease processes. Although its role in HFpEF remains an area of active debate, given that RDN works by decreasing renal and efferent sympathetic activity, the focus has more recently shifted from uncontrolled HTN to HFpEF, where it was posited that it could target the basic underlying pathophysiology of the disease.

The RDT-PEF study by Patel et al. was the only RCT evaluating the impact of RDN in HFpEF [40]. Although it demonstrated improvements in VO_2_ peak and E/e′ at 3 months, these benefits were not sustained, and the trial was prematurely terminated due to difficulties in patient recruitment [40].

Further observational studies by Kresoja et al., Rommel et al., and Zamani et al. were more encouraging, reporting reductions in NYHA class, biomarker levels, and improved echocardiographic parameters [41,42,51]. Additionally, Vogt et al. demonstrated significant and durable long-term reductions in HFA-PEFF score, primarily driven by improvements in morphological and biomarker subcategories with notable reductions in LVMI and BNP/NT-proBNP levels [48].

To better understand and contextualize the findings of these studies, it is essential to explore key sources of heterogeneity, including differences in HFpEF diagnostic criteria, RDN procedural techniques, follow-up durations, and overall study designs.

In the RDT-PEF by Patel et al., patients were prospectively enrolled and classified as having HFpEF based on NYHA class II-III symptoms, LVEF ≥50%, structural heart disease, elevated filling pressures, and heart failure hospitalization within the past 24 months [40]. In contrast, the studies by Kresoja et al. and Rommel et al. used retrospective classification based on the ESC 2016 guidelines. Their cohort was derived from patients who underwent RDN due to uncontrolled hypertension and were not primarily screened for heart failure symptoms [41,42]. Vogt et al., on the other hand, relied on the point-based HFA-PEFF scoring system to quantify the likelihood of HFpEF [48]. The study was initially designed to gauge the effects of RDN on blood pressure, as the concept of HFpEF was not as widespread; as a result, patients with HFA-PEFF scores ranging from 2 to 4 may have been misclassified. Zamani et al. defined HFpEF based on preserved ejection fraction (EF ≥ 50%) and a GLS > −18% assessed via CMR [51].

Variations in HFpEF definitions complicate cross-study comparisons and interpretation of RDN’s impact. For instance, a patient with mild HF symptoms, elevated BNP, and preserved EF, along with diastolic dysfunction on echocardiography, might qualify as having HFpEF in the studies by Kresoja et al. and Rommel et al., but would be excluded by the studies conducted by Patel et al. and Zamani et al.

In addition to diagnostic criteria, inconsistency in renal denervation techniques, such as catheter type, catheter modality, and completeness of ablation, vary across studies. In the RDT-PEF trial by Patel et al., RFA was performed using the Symplicity™ catheter, with a median of five successful ablations delivered to each renal artery [40]. Notably, 12 of the 17 patients had incomplete circumferential ablation due to anatomical challenges, and in the 5 patients who received complete denervation, this did not translate into superior clinical outcomes compared to those who did not [40].

In contrast, Kresoja et al. utilized both RFA in 115 patients using Medtronic systems and ultrasound-based ablation in 49 patients using the Paradise system. Rommel et al., utilizing the same patient cohort, reported that 45 patients underwent RFA and 15 underwent ultrasound RDN but did not specify catheter brands [41,42]. Vogt et al. used the Symplicity Flex^®^ catheter, similar to the RDT-PEF study; however, the procedure was conducted by a single operator, likely minimizing intraprocedural variability [48]. Neither Kresoja, Vogt, Zamani, nor Rommel commented on the procedural completeness of the ablation. Zamani et al. did not provide details regarding catheter type or method used. These intraprocedural inconsistencies and inconsistent reporting make it difficult to conclude whether variations in clinical results stem from the trials, therapeutic efficacy, or the extent of successful ablation.

Follow-up duration also varied widely across studies, ranging from 3 months to 9 years. Shorter follow-up intervals, as observed in studies by Patel et al., Rommel et al., and Zamani et al., may fail to capture delayed structural cardiac changes. Conversely, Vogt et al. was the only study with an extended 9-year follow-up. However, this study was limited by loss to follow-up, small sample sizes, and changes in medical therapy over time.

Substantial methodological variations also existed between the studies. All studies were observational in nature, with the RDT-PEF trial being the only RCT; however, due to stringent inclusion criteria and recruitment challenges, its sample size was only 25, making it susceptible to Type II errors and underpowered to detect whether RDN improved endpoints. Furthermore, the trial was not blinded and lacked a sham procedure, although outcome assessors were blinded to group assignment. Kresoja et al., Rommel et al., and Zamani et al. incorporated comparator arms; however, these were not randomized. Moreover, Zamani et al. included only six matched controls, limiting the strength of their comparison, and while notable for its extended follow-up, Vogt et al. lacked a control group. These design limitations, particularly the absence of randomization, increase the risk of confounding.

This is compounded by the fact that the therapeutic premise of RDN in HFpEF rests on an incompletely validated pathophysiologic model. The rationale for RDN in HFpEF is predicated on the assumption that SNS overactivity plays a central role in disease pathophysiology.

While conventional neurohormonal modulation has yielded variable results, RDN has been proposed as an alternative approach due to its selective action and preservation of chronotropic function, particularly in HFpEF phenotypes characterized by vascular stiffness and heightened sympathetic activity. However, this premise remains inadequately substantiated, as the evidence linking SNS overactivity to HFpEF has been inconsistent and primarily derived from surrogate endpoints such as plasma NE levels, impaired HRV, and increased MSNA. These are crude measures of sympathetic activity that can be influenced by several factors, including renal impairment. Additionally, while improvements in surrogate endpoints are promising, no study to date has demonstrated a benefit in hard clinical outcomes such as death or hospitalization.

While the current body of evidence remains limited, the presence of promising physiological improvements justifies the need for a robust RCT to further explore the potential therapeutic impact of RDN in HFpEF management and to identify which phenotypes of HFpEF, if any, would most likely benefit from RDN.

Currently, two ongoing RCTs aim to bridge this gap. The RDN-HFpEF trial seeks to enroll 200 patients with HFpEF who are on chronic maintenance medications, with 100 undergoing RDN and 100 undergoing a sham procedure. The primary objective is to assess changes from baseline in E/E at the 12-month follow-up. Similarly, the UNLOAD-HFpEF trial will evaluate the effects of RDN in 68 patients with HFpEF, focusing on hemodynamic outcomes such as exercise pulmonary capillary wedge pressure and endpoints, including NYHA class, biomarker levels, mortality rates, and heart failure hospitalizations.

## 6. Conclusions

The complex and multifactorial nature of HFpEF presents a significant challenge for physicians in optimizing management and developing new treatments for this debilitating chronic disease. RDN has emerged as a novel procedural intervention aimed at improving outcomes in select HFpEF phenotypes where sympathetic overactivation plays a more central role. Recent preclinical studies in mouse and rat models have demonstrated the beneficial effects of RDN, whereas human studies have shown mixed results.

Many investigations report statistically significant improvements in functional outcomes, cardiac biomarkers, and structural heart changes compared to controls; however, they are constrained by methodological shortcomings, including small sample sizes, heterogeneous diagnostic criteria, short follow-up durations, and a lack of randomization. Notably, none of these studies have demonstrated improvement in hard clinical outcomes such as mortality or heart failure hospitalizations.

Given these limitations, a clear need remains for a well-designed RCT to definitively determine whether the physiological improvements seen with RDN translate into meaningful clinical benefits, focusing on key endpoints such as mortality and heart failure hospitalizations.

## Figures and Tables

**Table 1:** Studies investigating the use of RDN in patients with HFPeF.

Author Name	Study Type	Cohort	Endpoints	Follow-Up Duration	Results
Patel et al., 2016 [40]	Randomized Controlled Trial	25 HFpEF patients (2:1 ratio RDN vs. OMT)	MLHFQ score, peak VO_2_, BNP, E/e′, LA volume index, LVMI, macro- and micro-vascular function, renal blood flow	12 months	No sustained improvement in primary endpoints; improvements in VO_2_ peak and E/e′ at 3 months in the RDT group, but this was not sustained at 12 months.
Kresoja et al., 2021 [41]	Observational Study	164 patients who underwent RDN (99 with HFpEF and 64 with uncontrolled AHT)	SVI, aortic distensibility, LV systolic/diastolic stiffness, NT-proBNP, E/e′, BPV, CPOI, PWV, NYHA class	6 months	Improvement in LV systolic and diastolic stiffness and reduced SBP, DBP, and NT-proBNP levels.
Rommel et al., 2023 [42]	Observational Study	60 patients with HTN who underwent RDN (30 HFpEF patients vs. 30 controls)	Pulsatile afterload, LV diastolic stiffness, SBP, DBP, BPV, NT-proBNP, E/E′, aortic distensibility, PWV	3 months	Improved NYHA class, decreased NT-proBNP levels, pulsatile left ventricular load, and arterial stiffness post-RDN.
Zamani et al., 2024 [51]	Observational study	22 patients with resistant HTN and HFpEF (16 underwent RDN vs. six received OMT	LVMI, GLS	6 months	Improvement in GLS at 6 months.
Vogt et al., 2024 [48]	Observational Study	70 patients who underwent RDN (22 patients with HFpEF)	E/E′, LA volume index, LVMI, HFA-PEFF score, BNP, SBP, DBP	9 years	Significant long-term reduction in LVMI and BNP/NT-proBNP in addition to reduced HFA-PEFF score.

## Data Availability

No new data were created or analyzed in this study. Data sharing is not applicable to this article.

## Abbreviations

The following abbreviations are used in this manuscript:

aHT	Arterial Hypertension
AL	Afterload
AD	Aortic Distensibility
BNP	B-type Natriuretic Peptide
BPV	Blood Pressure Variability
CMR	Cardiac Magnetic Resonance
CPOI	Cardiac Power Output Index
DBP	Diastolic Blood Pressure
DOAJ	Directory of Open Access Journals
E/e′	Ratio of Early Diastolic Transmitral Flow Velocity to Mitral Annular Early Diastolic Velocity
EF	Ejection Fraction
GLS	Global Longitudinal Strain
HFA-PEFF	Heart Failure Association Pre-test Assessment of HFpEF
HFpEF	Heart Failure with Preserved Ejection Fraction
HFrEF	Heart Failure with Reduced Ejection Fraction
HTN	Hypertension
KCCQ	Kansas City Cardiomyopathy Questionnaire
LAVI	Left Atrial Volume Index
LD	Linear Dichroism
LVEF	Left Ventricular Ejection Fraction
LVMI	Left Ventricular Mass Index
MLHFQ	Minnesota Living with Heart Failure Questionnaire
MDPI	Multidisciplinary Digital Publishing Institute
mos	Months
NT-proBNP	N-terminal pro-B-type Natriuretic Peptide
NYHA	New York Heart Association
OMT	Optimal Medical Therapy
PKG	Protein Kinase G
PWV	Pulse Wave Velocity
QOL	Quality of Life
RAAS	Renin–Angiotensin–Aldosterone System
RBF	Renal Blood Flow
RCT	Randomized Controlled Trial
RDN	Renal Denervation
RR	Relative Risk
SNS	Sympathetic Nervous System
SBP	Systolic Blood Pressure
SGLT2	Sodium–Glucose Co-Transporter 2
SVI	Stroke Volume Index
TGF-β	Transforming Growth Factor-beta
TLA	Three-Letter Acronym
VO_2_ peak	Peak Oxygen Consumption
